# Pre-Trained Deep Convolutional Neural Network for Clostridioides Difficile Bacteria Cytotoxicity Classification Based on Fluorescence Images

**DOI:** 10.3390/s20236713

**Published:** 2020-11-24

**Authors:** Andrzej Brodzicki, Joanna Jaworek-Korjakowska, Pawel Kleczek, Megan Garland, Matthew Bogyo

**Affiliations:** 1 Department of Automatic Control and Robotics, AGH University of Science and Technology, 30-059 Kraków, Poland; pkleczek@agh.edu.pl; 2 Cancer Biology Program, Department of Pathology, Stanford University School of Medicine, Stanford, CA 94305, USA; garlandm@stanford.edu (M.G.); mbogyo@stanford.edu (M.B.); 3 Department of Microbiology and Immunology, Stanford University School of Medicine, Stanford, CA 94305, USA; 4 Department of Chemical and Systems Biology, Stanford University School of Medicine, Stanford, CA 94305, USA

**Keywords:** clostridioides difficile, fluorescence images, image analysis, classification, deep neural networks, convolutional neural networks, transfer learning

## Abstract

*Clostridioides difficile* infection (CDI) is an enteric bacterial disease that is increasing in incidence worldwide. Symptoms of CDI range from mild diarrhea to severe life-threatening inflammation of the colon. While antibiotics are standard-of-care treatments for CDI, they are also the biggest risk factor for development of CDI and recurrence. Therefore, novel therapies that successfully treat CDI and protect against recurrence are an unmet clinical need. Screening for novel drug leads is often tested by manual image analysis. The process is slow, tedious and is subject to human error and bias. So far, little work has focused on computer-aided screening for drug leads based on fluorescence images. Here, we propose a novel method to identify characteristic morphological changes in human fibroblast cells exposed to *C. difficile* toxins based on computer vision algorithms supported by deep learning methods. Classical image processing algorithms for the pre-processing stage are used together with an adjusted pre-trained deep convolutional neural network responsible for cell classification. In this study, we take advantage of transfer learning methodology by examining pre-trained VGG-19, ResNet50, Xception, and DenseNet121 convolutional neural network (CNN) models with adjusted, densely connected classifiers. We compare the obtained results with those of other machine learning algorithms and also visualize and interpret them. The proposed models have been evaluated on a dataset containing 369 images with 6112 cases. DenseNet121 achieved the highest results with a 93.5% accuracy, 92% sensitivity, and 95% specificity, respectively.

## 1. Introduction

*Clostridioides* (formerly *Clostridium*) *difficile* infection (CDI) causes diarrhea, nausea, pseudomembranous colitis and dehydration, which, in some cases, leads to death. It is one of the most common causes of nosocomial infections, where patients can have multiple risk factors for disease, including advanced age and severe underlying disease [1,2]. Recurrent episodes occur in 10–25% of patients [2]. A rise in CDI has been observed in recent years, with incidence nearly doubling in the United States (US) from 2001–2010 [3,4,5]. In 2011, the US had an estimated 453,000 cases, and CDI was associated with approximately 29,000 deaths [6,7]. Similar trends are occurring in other countries [1,8]. Further studies assessing infection rates from 2010–2015 confirm that CDI remains a serious public healthcare burden costing US$5.4–6.3 billion per year [9,10].

A major risk factor of CDI is broad-spectrum antibioticotherapy which causes havoc in the body and allows *Clostridium difficile* bacteria to multiply quickly [1,2]. Although the typical way of dealing with severe cases of CDI is with other, dedicated antibiotics, a wrongly matched or ineffective antibiotic will only create a more favorable environment for the bacterial growth. Moreover, a new virulent strain of *C. difficile*, exhibiting greater toxin production and resistance, has emerged in the last decade [11]. There is therefore a need to develop new methods of CDI treatment—such as novel therapies focusing on directly neutralising the effects of *C. difficile* toxins, instead of killing the bacteria [12]. However, developing a new drug requires extensive testing, which is often carried out manually, making it a slow, tedious and unreliable process [13]. In order to improve it, microscopic images need to be automatically checked, individual cells segmented and then classified as either dead or alive by an algorithm.

Computer image processing has progressed rapidly in the last decade, thanks to the invention of deep convolutional neural networks (CNN) and sophisticated processing and visualization algorithms [14]. Deep learning in general is currently one of the most popular methods from the broad artificial intelligence group and is used in many yet unsolved challenges. In particular, CNNs can be applied in a variety of image processing tasks including pattern recognition, segmentation and classification problems [15]. Their key advantage is good automatic extraction and learning of image features, which is a very time and computationally consuming stage. The development of hardware capabilities and access to large databases like the Image Large Scale Visual Recognition Challenge (ILSVRC) dataset has allowed the creation of very well-performing neural networks. Modern general-purpose computing on graphics processing units (GPGPUs) make parallel computing possible and shorten the training time on those very large datasets. Furthermore, the idea of transfer learning succored by data augmentation have made it possible to use this accumulated knowledge in more specific tasks, where only a limited amount of training data is often available (both of these approaches are discussed in further sections).

In this study, we focus on automatic fluorescence image analysis using deep neural networks to identify potential drug candidates based on automated analysis of the morphological changes seen in human fibroblast cells secondary to the cytotoxic action of *C. difficile* toxins.

The goal of this algorithm is to separate individual cells based on fluorescence images and classify each cell as adherent or rounded. The main novelty of our research stems from the fact that the proposed algorithm can be used as an automatic and objective scheme to screen drugs—we assured its automation and objectivity by:

- proposing a solution for cell classification of fluorescence images based on the CNN models, which—thanks to using data augmentation and transfer learning—yielded satisfactory results even for a limited amount of data (we deployed and compared four pre-trained CNN architectures, including VGG-19, ResNet50, Xception, and DenseNet121 with an adjusted, densely connected classifier),
- removing unnecessary objects from the background of the image by an inpainting technique,
- training the network on different types of images and adding regularization in order both to make the model resistant to cell staining deviations and to avoid the overfitting problem.

### 1.1. Fluorescence Images

Fluorescence images are made by adding natural or artificial components to cells, which, under a light source, glow in a specific way. Different kinds of fluorescence markers have different properties. Two of them, green fluorescent protein (GFP) and 4’,6-diamidino-2-phenylindole (DAPI), were used in this research.

**Figure 1:**
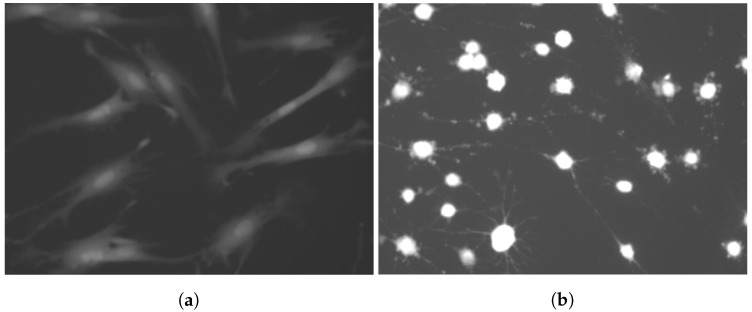
Green fluorescent protein (GFP) staining used to observe morphological features: (**a**) shows living cells, (**b**) dead ones.

Green fluorescent protein (GFP) is a common marker used to observe whole-cell features. Changes in colour, shape and size, after exposure to toxins, can be visualised. Long, adherent cells are their natural, healthy state. After damaging the cytoskeleton, fibroblast cells shrink and become brighter and more rounded (Figure 1). These features are considered to be particularly important in their classification.

**Figure 2:**
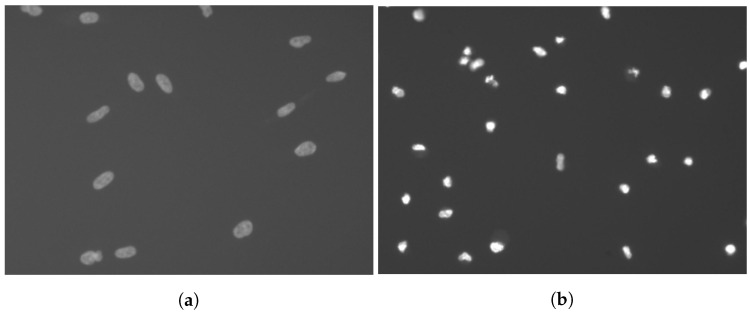
4’,6-diamidino-2-phenylindole (DAPI) images, which show only cell nuclei, are used for nuclei detection and cell separation: (**a**) shows nuclei of living cells, (**b**) dead ones.

4’,6-diamidino-2-phenylindole (DAPI) is a compound that reacts with DNA. It can be used to mark cell elements such as nuclei (Figure 2). Because each observed cell has only one nucleus, these images help to distinguish overlapping cells.

### 1.2. Related Works

Although there are many works about different CDI treatments and about predicting CDI based on clinical data using machine learning methods [16,17], to the best of our knowledge there are almost no research reports about deploying computer image processing methods for *C. difficile* cell segmentation in conjunction with machine learning methods for the classification of these cells (the segmentation method proposed by Memariani and Kakadiaris [18] is designed for a different modality, namely for scanning electron microscopy). In our previous research [13] we proposed a preliminary solution for the assessment of cells; however, we observed erroneous results for cells that had irregular borders, uneven intensity, and protruding lines. The following algorithm has been implemented including pre-processing stage and classification part. Firstly, we adjusted the contrast for both DAPI and GFP images. Then, we localized cell nuclei on DAPI images. We segmented them, indexed them, and used their position to track whole cells on corresponding GFP images. Finally, single cell windows, extracted from GFP images, were used to train a naive Bayes classifier, which had an accuracy of 91%, sensitivity of 93%, and specificity of 91% respectively [13]. Furthermore, open source image processing programs like ImageJ contain different plugins like *Fiji*, *Ice* or *BioVoxxel Toolbox* to calculate the cells; however, these are based on a permanent interaction with the user or manual selection.

## 2. Methods

The results of our previous study indicated that the preprocessing stage, which consists of splitting overlapping cells, plays a crucial role in the classification task. Although our preliminary solution gave promising results (see paper [13]), the statistical outcomes while using the application still did not meet our expectations. We demonstrate that, for many images, especially with unclear borders and inhomogeneous areas, the algorithm was not complex enough to solve the problem.

To address the limitations of the initial algorithm, we utilized pre-trained convolutional neural networks, as deep learning models tend to perform well with medical computer vision tasks. The aim of the pre-trained network is to reuse a complex deep learning model with already calculated weights to classify cells into two categories. Classification into adherent or rounded cells is based on previously preprocessed images.

**Figure 3:**
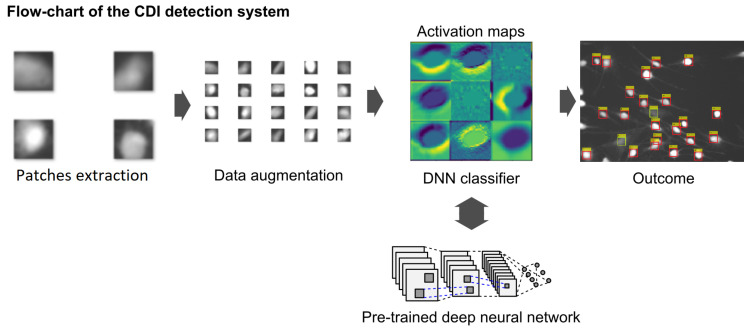
Algorithm stages for the classification of cells: image preprocessing consisting of nuclei and cell detection, cell separation, patch extraction, dataset augmentation, pre-trained convolutional neural network (CNN) architecture adjustment, comparison and evaluation.

The solution is divided into four stages (Figure 3): image preprocessing (cell detection), dataset augmentation, pre-trained CNN architecture comparison, and evaluation. The general procedure described in this paper is based on our solution proposed in [13]. However, at each stage we introduce a number of significant modifications meant to improve its effectiveness.

Cell detection and data augmentation stages were performed using Matlab software, especially the Image Processing Toolbox [19]. For deep learning, classifier training, comparison and evaluation we used Python programming language, with the up-to-date Keras library [20].

### 2.1. Cell Detection

Despite the fact that CNN-based architectures are implemented and adapted to detect and segment multiple objects occurring in the input image, in our solution, we propose to first detect and crop cells and afterwards perform the classification stage. This approach is based on our initial attempts (described in paper [13]), which showed that, for our specification, the classification results were insufficient.

**Figure 4:**
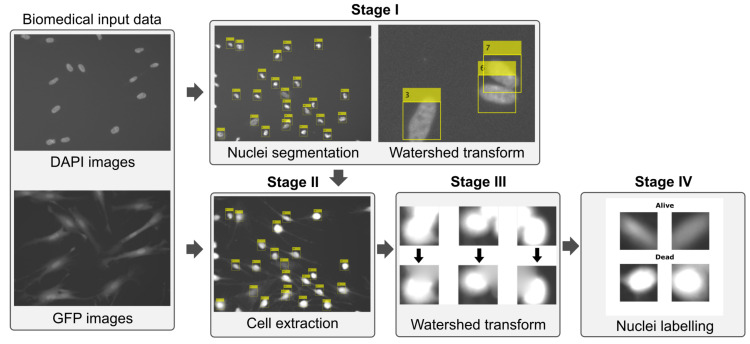
Flowchart of the pre-processing stage based on watershed transform (required to create individual cell patches using DAPI and GFP images).

To localize and segment CDI cells, we combined the information gained from both DAPI and GFP images—cells’ nuclei are firstly identified and localized using DAPI images and then whole cells are extracted from GFP images based on nuclei position and foreseen size (see Figure 4).

The initial step of the cell detection stage consists of two parts: nuclei segmentation and separation of partially overlapping objects. Firstly, we perform image binarization using adaptive thresholding (based on Bradley’s method, proposed in 2007 [21]) followed by area filtering.

In adaptive binarization, the threshold is determined for each individual pixel, based on its neighborhood, where the neighbourhood can be of any size. To calculate the surroundings, we used the default values [21]:

(1) $$\begin{array}{ccc} \; & h_{n}=2\frac{h_{i}}{16}+1 & w_{n}=2\frac{w_{i}}{16}+1, \end{array}$$

where $h_{i},h_{n},w_{i},w_{n}$ are the image and neighborhood height and width, respectively, whereas the area threshold value of 100 px is based on medical knowledge.

**Figure 5:**
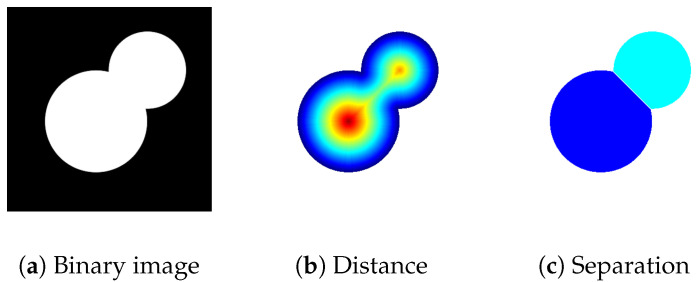
Watershed transform is used to split overlapping cells: (**a**) binary image, (**b**) calculated distance transform, (**c**) afterwards, each pixel is assigned to one of the objects based on belonging to one of the catchment basins.

Next, we separated overlapping nuclei using watershed transform, a technique that draws inspiration from the behavior of water. This method is applied to a binarized DAPI image. For each pixel of each object, a distance transform (i.e., distance to the nearest background pixel) is calculated. The resulting image is displayed as a surface representing high and low elevations. Local minima are considered catchment basins—points towards which the imaginary water flows. Each pixel is assign to one of the catchment basins based on the path of the steepest slope. Borders between watershed regions are called ridge lines. We use these lines to separate overlapping cell nuclei (Figure 5).

There are several definitions of watershed transform, based on a function used to describe water flow, both of which lead to different algorithm behaviors. We used the method proposed by Fernand Meyer in [22], which was provided in Matlab Software. A detailed review of these variants can be found in [23].

**Figure 6:**
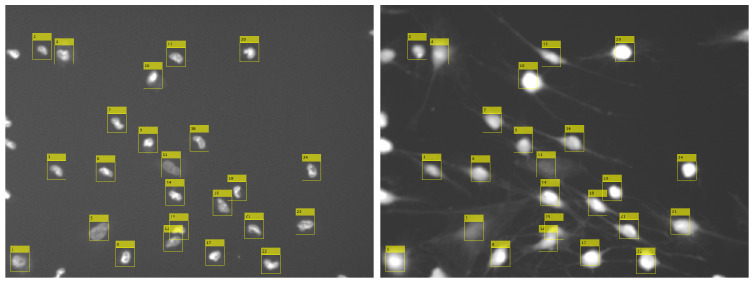
Cell localization, gained from DAPI image processing (**left**), is applied to a corresponding GFP image (**right**).

For every single nucleuss the coordinates of its centroid are computed using the following equations:

(2) $$\begin{array}{cc} \; & x_{c}=\frac{\sum_{i=1}^{h}\sum_{j=1}^{w}k}{A_{O}}\;k=\left\{\begin{array}{cc} i & \mathrm{if}\;\mathrm{pixel}\;(i,j)\in O\\ 0 & \mathrm{otherwise}\; \end{array}\right.\\ \; & y_{c}=\frac{\sum_{i=1}^{h}\sum_{j=1}^{w}l}{A_{O}}\;l=\left\{\begin{array}{cc} j & \mathrm{if}\;\mathrm{pixel}\;(i,j)\in O\\ 0 & \mathrm{otherwise}\; \end{array}\right. \end{array}$$

where $x_{c},y_{c}$ are centroid coordinates, h,w are image dimensions, *O* is the measured object, and $A_{O}$ is object’s area (number of pixels). The centroid coordinates are then used to extract the corresponding GFP image, which is a 64×64 px patch containing that nucleus (the patch is subsequently saved to a separate file). We ignore nuclei whose centroids are located at a distance of less than 32 px from the image borders as significant cell fragments that are outside the microscope area, thereby enabling correct classification. Figure 6 shows the results of the steps carried out so far.

**Figure 7:**
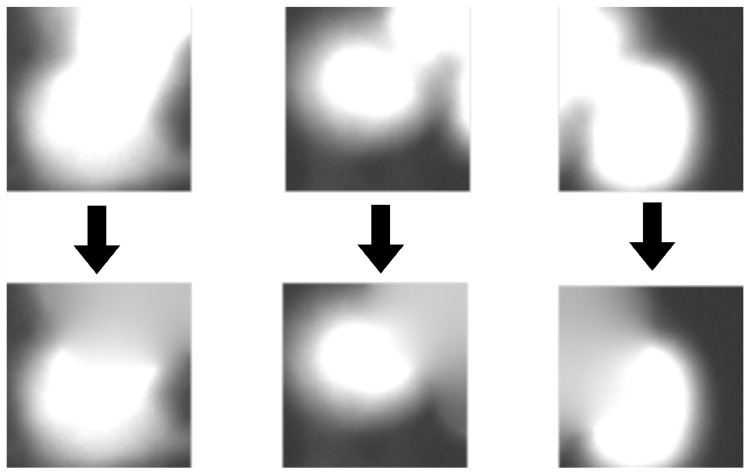
Image borders with fragments of other cells (**top row**) are cleared using watershed transform and inpainting (**bottom row**).

Patches extracted during the previous stage contain not only the desired cells, but also parts of neighboring objects. In order to perform an effective classification process, we have to inpaint those areas. We achieve this by using watershed transform for the second time, this time on GFP images, to separate the main cell from surrounding cells. Afterwards, we automatically binarize those images using the Otsu method [24] and apply the same workflow as described before for DAPI separation (see Figure 5). After separation, a cell in the center remains unchanged while others are blended into the background. The algorithm used for inpainting starts with pixels on the borders and smoothly interpolates inward. It calculates the discrete Laplacian over a given mask and solves the Dirichlet boundary value problem. The results of the clearing procedure are presented in Figure 7.

### 2.2. Dataset Augmentation

Human foreskin fibroblasts were cultured in controlled conditions, according to the procedure described in [13], and then imaged on a BioTek Cytation 3 plate reader in DAPI and GFP channels.

**Figure 8:**
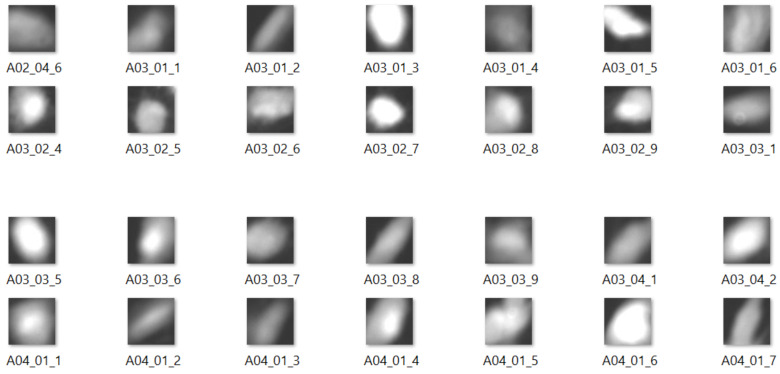
Sample images in the preprocessed dataset.

The dataset consists of 369 cases, each with two images of the same cell—one taken using DAPI and another using the GFP staining technique. In the cell detection stage, we extracted 6112 patches of individual cells in total, each of 64×64 px (Figure 8).

Since supervised learning methods, such as convolutional neural networks, require samples to be labeled, cells were manually assigned to one of two categories—either adherent or rounded. This assignment was checked independently by an expert. As there were twice as many rounded cells (3965 samples) as adherent (2122 samples), we decided to deal with this class imbalance problem [25]. We oversampled the “adherent” class using basic image transformations (such as rotation and mirror reflection), applied in a random fashion. The final dataset consisted of 8208 pictures distributed between two roughly equinumerous classes. This was later split into train, test and validation subsets, containing 60%, 20% and 20% of samples, respectively.

### 2.3. Deep Neural Networks with Transfer Learning

Both the amount and quality of data need to be considered when developing a deep learning framework for medical problems [26]. Gathering samples might in some cases be very expensive or even impossible. To enable deep learning architectures to be deployed in small dataset problems, a method called transfer learning has been proposed. Instead of collecting data, it shares a model which has already "seen" a lot of varied and very advanced data before. It is a concept that assumes that models pre-trained on a very huge datasets are able to generalize and solve a wide variety of problems. Parts of those ready-made, convolutional neural networks may be reused and only some of the top layers need to be retrained for a specific task. They are widely used with great efficiency in many fields like computer vision, signal processing, and natural language analysis.

Using fragments of a pre-trained model for a completely new task, without training the whole network again, is possible thanks to CNN’s specific properties, such as gradual feature extraction in subsequent layers. Lower layers typically detect common patterns like lines and edges, the middle find parts of objects, while the last ones learn to recognize full objects, in different shapes and positions [27]. The main concept is to use initial layers from a pre-trained model and retrain only the final few on new images [28]. The formal definition of transfer learning is formulated in terms of a domain and a task. Given a source domain $D_{S}$ and learning task $T_{S}$, a target domain $D_{T}$ and learning task $T_{T}$ transfer learning aims to improve the learning of the target predictive function $f_{T}\left(\cdot \right)$ in $D_{T}$ using the knowledge in $D_{S}$ and $T_{S}$, where $D_{S}\neq D_{T}$, or $T_{S}\neq T_{T}$ [29].

**Figure 9:**
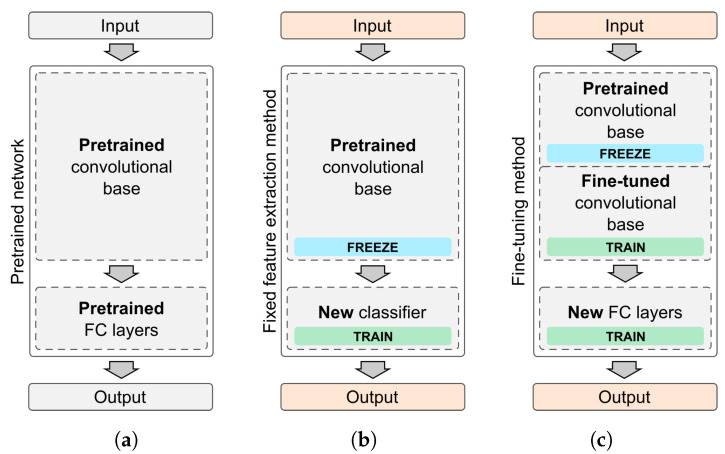
There are three transfer learning scenarios: (**a**) train whole model, (**b**) apply a new classifier on top of a pre-trained convolutional base, (**c**) fine-tune the base, by re-training one or more convolution layers.

To re-purpose a pre-trained model we have to apply one of three methodologies (Figure 9):

- Train the entire model—use the implemented architecture of the pre-trained model and train it on your dataset. Instead of using random weights, start from values of a pre-trained model.
- Feature extraction (freezing CNN model base)—train a new classifier on top of the pre-trained base model. The weights of convolution layers are left unchanged and only the last, fully connected layer is trained.
- Fine-tuning (training also some convolution layers)—retrain one or more convolution layers in addition to a fully connected classifier. Original convolution layer weights are used as starting points. Unlocked convolution layers are only tuned to a new problem.

Deep learning models’ performance increases proportionally to the amount of data [30]. Therefore, most architectures have to be trained on very large datasets, which, typically, are not readily available. However, the most popular models are provided on an open source basis, with pre-trained weights. They are trained on the ImageNet dataset [31]—a collection of 14,197,122 images from 21,841 real-life categories. This dataset is used in worldwide competitions, from which, every year, better algorithms emerge. We briefly describe VGG, ResNet, Inception, and DenseNet architectures and present a comparison of their performance in *Clostridium difficile* cytotoxicity classification.

### 2.4. Pre-Trained Deep Learning Architectures

In recent years, the ImageNet Large Scale Visual Recognition Challenge (ILSVRC) has been dominated by state-of-the-art CNNs and deep learning techniques, including pre-trained networks. Starting from the famous AlexNet in 2012 [32], more advanced architectures such as VGG-19, ResNet50, Xception, and DenseNet121 represented some of the highest performing techniques over the past few years [33].

These models, alongside pre-trained weights and useful functions, are provided by the Keras library [20].

**Figure 10:**
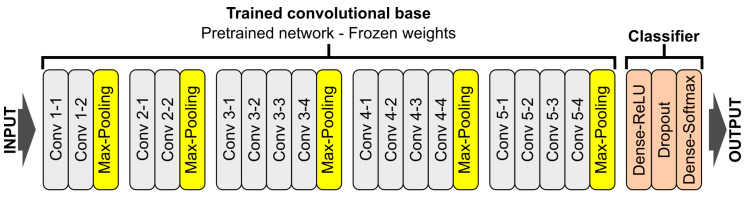
Schematic overview of the personalized VGG-19 network architecture with description of layers.

**VGG16 and VGG19** [34]—one of the simplest architectures consisting of only 3×3 convolutional layers stacked on top of each other. Reducing volume size is handled by max pooling (Figure 10).

**Figure 11:**
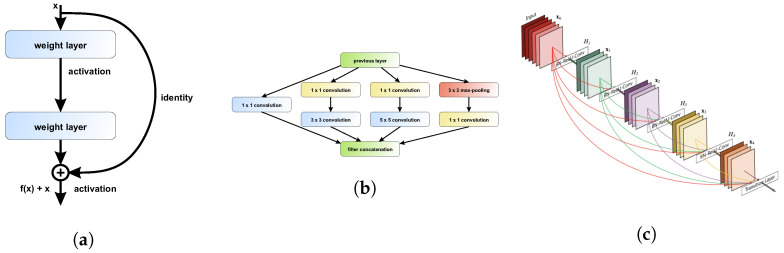
CNN-based neural network architecture element: (**a**) a sample residual block explaining the idea behind ResNet model [35]; (**b**) the general idea of Xception network [36]; (**c**) schematic diagram of DenseNet architecture [37].

**ResNet** [35]—unlike traditional sequential networks like VGG, ResNet presents a network-in-network architecture. The ResNet model is built from layers stacked on top of one on another and shortcuts at each layer that connect the input of that block with the output. Thanks to the proposed solution, consisting of an-Identity Blocks and b-Convolutional Blocks, it was possible to implement a very deep neural network with skipping-connections that helped to address the vanishing gradient problem (Figure 11a).

**Inception** [38]—in this model, layers are often connected in parallel instead of being stacked on top of one on another (Figure 11b). Complex filters are separated into various simple ones. One of the modifications, called Xception [39], uses depthwise separable convolutions and 1×1 pointwise convolutions to reduce feature map dimensions [40,41].

**DenseNet** [37]—the latest network, where every layer is connected to all previous ones. Features are calculated on the basis of information extracted at all previous stages (Figure 11c).

One of the crucial components of CNN architectures are activation functions that are responsible for the output of a single neuron, and also the accuracy and computational efficiency of the training model [42,43].

The activation function of a node defines the output of that node given a set of inputs. In our research, we take advantage of nonlinear activation functions, including sigmoid and Rectified Linear Unit (ReLU), which allow the CNN architecture to create complex mappings of knowledge between the network’s inputs and outputs and are essential for learning complex data boundaries. The sigmoid function, which is also called logistic, is monotonic and differentiable, with an output range between [0,1]. It can be described using Equation (3):

(3) $$\mathrm{sigmoid}\left(x\right)=\frac{e^{x}}{e^{x}+1}$$

As well as advantages such as gradient smoothing and clear predictions, it has a few main weaknesses, including the fact that sigmoid outputs are not zero-centered, the vanishing gradient problem, where weights in lower layers are virtually unchanged, as well as expensive computation. One of the most widely used functions due to its many advantages is the Rectified Linear Unit (ReLU) activation function, or ReLU for short, which is a piecewise linear function defined as the positive part of its argument. The regular version is given by Equation (4):

(4) $$\mathrm{ReLU}\left(x\right)=\left\{\begin{array}{cc} 0 & x\;<\;0\\ x & x\;\geq \;0 \end{array}\right.$$

where *x* is the input to the neuron. Rectified Linear Units, compared to sigmoid functions, allow faster and effective training of deep neural architectures and complex datasets. Furthermore, different versions including leaky or parametric ReLU have been proposed and achieve higher results for certain problems. ReLU has many advantages: it reduces the number of active neurons due to a zero in the negative domain, as well as not saturating, is very computationally efficient, converges much faster than other methods, and prevents the vanishing gradient problem.

In the final layer, we use the Softmax activation function, which is a common choice for the last layer in most state-of-the-art architectures to normalize the output of a probability distribution over predicted output classes that sum to one. The Softmax function is a generalization of the logistic function to multiple dimensions. The function’s role is to normalize the output between 0 and 1. This can be interpreted as “how sure the network is of each class”. It can be used in both binary and multi-class problems, provided that each object belongs to one class. The Softmax function is given by Equation (5):

(5) $$\mathrm{Softmax}\left(y_{i}\right)=\frac{e^{y_{i}}}{\sum_{j=0}^{K}e^{y_{i}}}$$

where $y_{i}$ are the elements of the input vector to the Softmax function, $e^{y_{i}}$ is the standard exponential function applied to each element of the input vector, and $\sum_{j=0}^{K}e^{y_{i}}$ is the normalization term, which ensures that all the output values of the function will sum to 1 and each will be in the range (0, 1).

In order to reduce the dimensions, we applied max pooling layers, which calculate the maximum for a given part of the feature map. They also make the network invariant to small changes in the input and reduce its complexity [28].

According to the transfer learning methodology, we leave most layers from one of the mentioned models unchanged and add a dedicated classifier on top of the base. The classifier consists of fully connected layers, a dropout layer (which randomly cuts off some connections to reduce the overfitting problem) and a Softmax layer for the classification task [42,44].

### 2.5. Parameter Selection

The necessary condition for proper neural network training is the correct selection of hyperparameters. To achieve this, we performed grid search optimisation, with 5-fold cross-validation on the training dataset. We tested ReLU, sigmoid, TanH and linear activation functions for fully (densely) connected layers in the classifier. The dropout rate, which determines the fraction of the input units to drop, varied between 0.3 and 0.7. The parameters that gave the most promising results are presented in Table 1.

Training hyperparameters included: the optimizer type (SGD, RMSprop, Adagrad, Adadelta, Adam, AdaMax, Nadam), the batch size (32, 64, 128, 256, 512) and the number of epochs (5, 10, 15, 20, 30). In the table, we present the parameters that achieved the highest accuracy (Table 1). As each optimizer works best with a different learning rate, we decided to stick to default values set in Keras [45].

In our implementation, the AdaMax optimizer, which is a modification of a popular optimization algorithm called Adam, gave the highest results. As stated in [46], the AdaMax algorithm computes individual adaptive learning rates for different parameters from estimates of the first and second moments of the gradients. The AdaMax variant is based on the infinity norm, instead of $∥L^{2}∥$. This generalised version is simpler and more stable.

**Figure 12:**
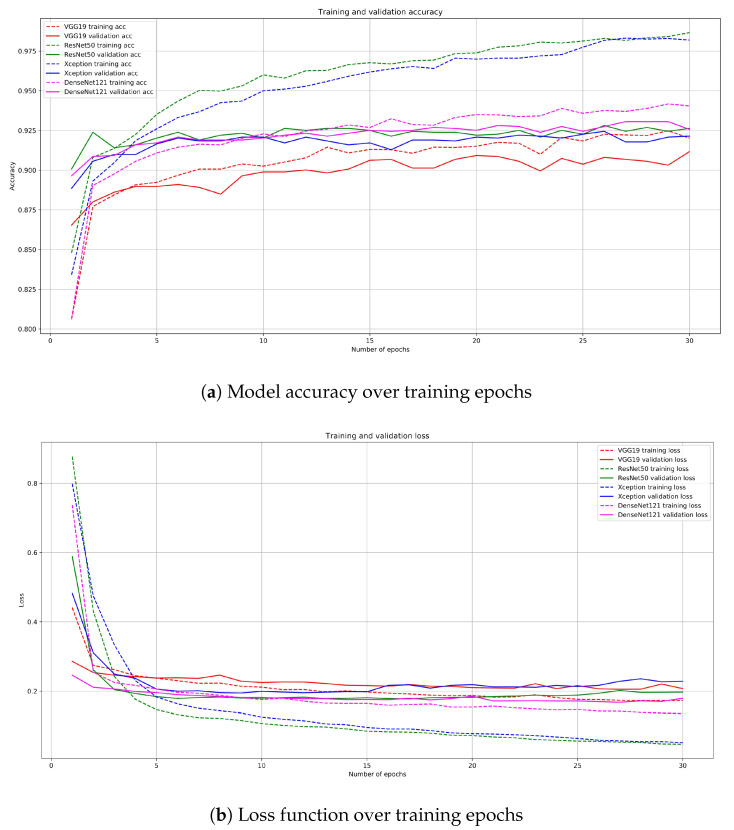
Line plot of: (**a**) classification accuracy and (**b**) loss function—over training epochs when optimizing the model.

By checking the accuracy and loss of training and validation sets, we were able to control the model’s performance during training. Figure 12 shows that the ResNet50 and Xception models overfit the data—in each case, their training accuracy increases, while the validation accuracy remains the same. On the other hand, DenseNet architecture slowly improves with each epoch, leading to a better final result.

In the end, we trained all four network models 5 times, with optimal parameters. The final results were verified on a separate test set and averaged.

## 3. Results

### 3.1. Visualization of the Convolutional Layers

**Figure 13:**
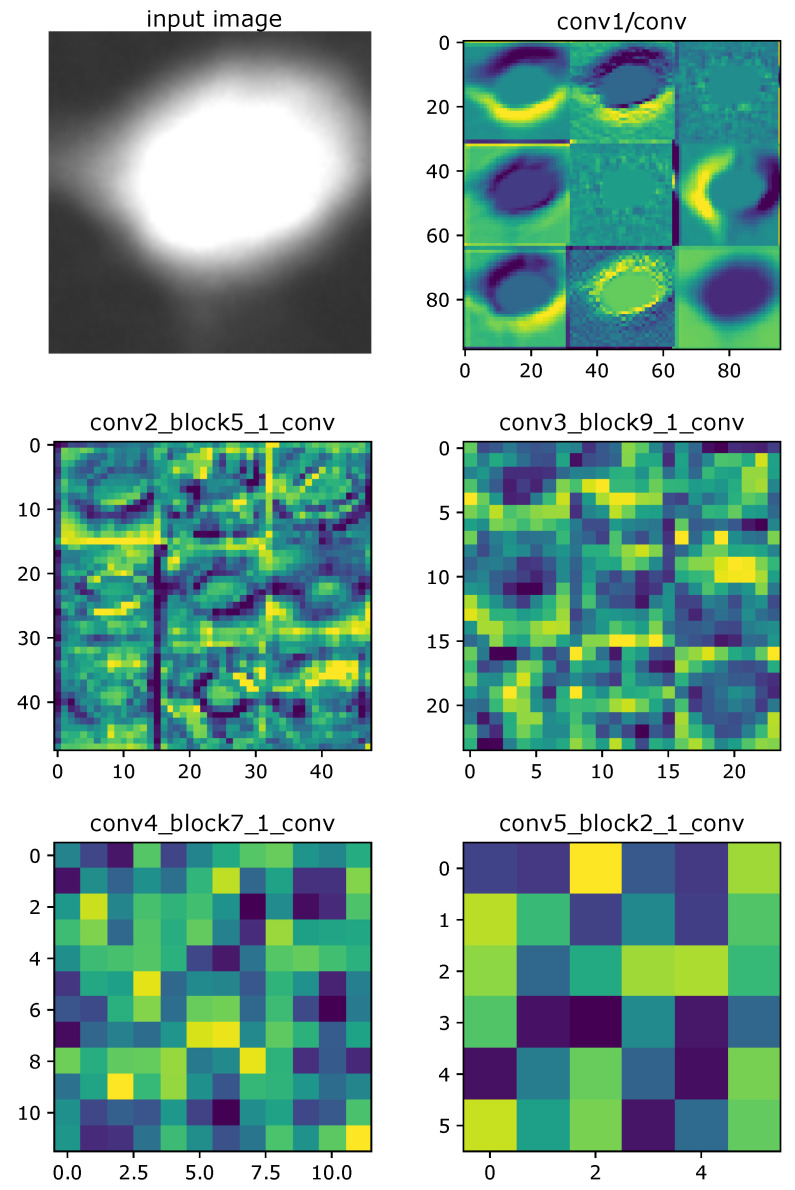
Activations of sample convolution layers in ResNet121 model. In early layers, cell edges are highlighted. In the middle, important sections are colored. The final layers are unrecognisable squares.

Artificial neural networks are commonly described as black boxes [47]. They adjust weights and find patterns themselves, based on learned samples. It is therefore sometimes hard to define precisely why the network chooses a particular result, which is seen as a vast weakness of these methods, especially in the medical sector [48]. However, by visualizing its behavior step by step, in each individual layer, CNN networks become more interpretable and explainable [49]. Furthermore, we are able to confront this knowledge with an expert to confirm that the network is working properly while comparing the area of interest for the classification task. In this task, we analyse whether the activation and heat maps reach high values in the region of interest, which is the cell itself. We can observe in the first layers of the activation maps (Figure 13) that the cell has been correctly identified and the classification process is based on this area.

Figure 13 presents sample activations of five different convolution layers in the DenseNet121 model. By analysing these images, we see which features are important for the network. For example, in early layers, cell edges are marked in yellow. The deeper we go, the less recognisable an object becomes. In the middle layers, large areas are colored. The final areas are only squares—those marked with the brightest colors suggest important sections [50].

**Figure 14:**
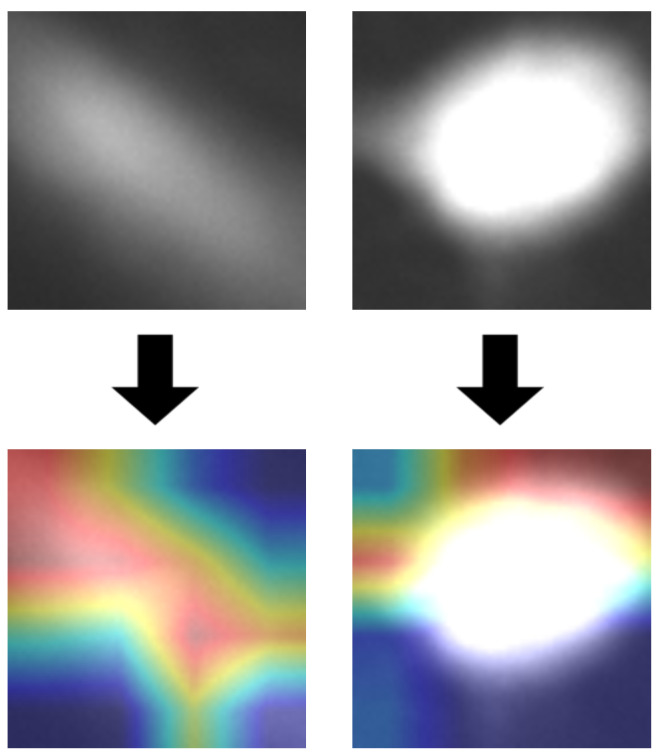
Heat maps visualising response of the last layer of VGG-19 model, superimposed on input images.

To show which parts of the original image were used for classification, we superimposed the activation of the final convolution layer on the input image, thus creating a heat map [50]. In Figure 14, we can observe that the network correctly recognised object shapes. We can also confirm, that, in the second example, background was more important than the object itself, which is an undesirable behavior.

### 3.2. Statistical Analysis

Statistical analysis of classification tasks implies the use of several terms and concepts. For the quality analysis, we considered living cells as positive (P), and dead ones as negative (N). The combination of these symbols helps calculate the indicators most commonly used in binary classification problems, including accuracy, precision, sensitivity, and specificity. Accuracy refers to the closeness of a measured value to a known value, specified as the percentage of correctly classified samples:

(6) $$\mathrm{ACC}=\frac{\mathrm{TP}+\mathrm{TN}}{P+N}$$

Precision refers to the closeness of two or more measurements to each other and is specified as the percentage of correctly classified living cells among all cells considered alive:

(7) $$\mathrm{PREC}=\frac{\mathrm{TP}}{\mathrm{TP}+\mathrm{FP}}$$

Sensitivity, also called recall or the true positive rate (TPR), is described as the percentage of correctly classified living cells among all living cells:

(8) $$\mathrm{SENS}=\frac{\mathrm{TP}}{\mathrm{TP}+\mathrm{FN}}$$

Specificity, also referred to as the true negative rate (TNR), is described as the percentage of correctly classified dead cells among all dead cells:

(9) $$\mathrm{SPEC}=\frac{\mathrm{TN}}{\mathrm{TN}+\mathrm{FP}}$$

$F_{1}$ score, also known as the balanced F-score or F-measure, can be interpreted as a weighted average of the precision and sensitivity:

(10) $$F_{1}=\frac{2*\mathrm{PREC}*\mathrm{SENS}}{\mathrm{PREC}+\mathrm{SENS}}$$

The comparison of the performance of different models is presented in Table 2. The best one proved to be the recently popular DenseNet121, achieving 93.3% accuracy, and equally high sensitivity and specificity. Other architectures were only slightly worse in terms of their accuracy, with ResNet50 achieving (after twice as few learning epochs) 92.6% accuracy.

### 3.3. Comparison with Other Research Works

Finally, we compared the achieved results with our previous paper [13]. We used the same image dataset as a reference point. The new solution, based on deep learning methods with transfer learning, achieved better generalization for the whole dataset. It could be observed that, for challenging cases, the classification process was stable contrary to classical machine learning algorithms, including the naive Bayes classifier (Table 3).

## 4. Conclusions and Discussions

These results demonstrate that the latest pre-trained models, including VGG-19, ResNet50, Xception and DenseNet121, have the potential to be deployed in medical imaging domains, including image processing and analysis. CNN models can take medical imaging technology further and improve its capabilities, providing a higher level of automation while speeding up processes and increasing productivity.

The algorithm in this study achieved 93.5% accuracy, and equally high sensitivity and specificity, which is a state-of-the-art result in *C. difficile* cytotoxicity classification. We have proven that transfer learning may be useful for many different challenging tasks and is one answer to computer vision problems, for which often only small datasets are available. Medical applications prove that sophisticated CNN architectures have the ability to generalize and learn very recherché features and not only map knowledge on images similar to those from the ImageNet database, but also correctly classify very different cases.

The weakest point of this algorithm is the necessity of manually labeling data. As a result, the network might inherit some faults from an analyst, as correct judgement of a cell is, in many cases, difficult even for a human. One way to mitigate this limitation would be to use a bigger dataset, labeled by a larger group of experts.

### Future Work

In this research, we have described a deep learning-based architecture for objective drug screening; however, the idea was based on deploying the transfer learning methodology. As this confirmed the correct direction for the classification of objects in fluorescence images, we propose a few directions. Future research will mainly focus on the enlarging of the dataset, which will enable the use of transfer learning with the fine-tuning of the last layers. The best performing architecture—DenseNet121—should be retrained with one or more convolution layers unlocked for training (as described in Methods—Section 2.3).

In the next step, based on an increased and augmented database, we will implement our own dedicated architecture and train it from scratch.

## Figures and Tables

**Table 1:** Hyperparameters and training setings for the CNN models.

Model	Dropout	Activation Function	Optimizer	Epochs	Batch
VGG-19	0.7	sigmoid	AdaMax	30	128
ResNet50	0.5	ReLU	AdaMax	20	512
Xception	0.5	ReLU	AdaMax	20	512
DenseNet121	0.5	sigmoid	AdaMax	30	256

**Table 2:** Model performance [%].

Model	ACC	PREC	SENS	SPEC	F1
VGG-19	91.7	92.0	90.8	92.5	91.3
ResNet50	92.6	94.2	91.1	94.2	92.6
Xception	92.0	92.6	90.7	93.2	91.6
**DenseNet121**	**93.3**	**94.0**	**91.8**	**94.5**	**93.0**

**Table 3:** Comparison with article [13] [%].

Classifier	ACC	SENS	SPEC
Naive Bayes	92.6	93.0	91.0
**DenseNet121**	**93.3**	**91.8**	**94.5**
